# *Clostridium septicum* Empyema in an Immunocompetent Woman

**DOI:** 10.1155/2010/231738

**Published:** 2010-05-16

**Authors:** Alexander B. Granok, Patrick A. Mahon, Genesio W. Biesek

**Affiliations:** ^1^Infectious Disease Associates, 399 Daniel Webster Highway, Merrimack, NH 03054, USA; ^2^Surgical Care Group, PC, 87 McGregor Street, Suite 3100, Notre Dame Pavilion, Manchester, NH 03102, USA; ^3^Manchester Internal Medicine Associates, 1650 Elm Street, Suite 302, Manchester, NH 03101, USA

## Abstract

We report a case of a *Clostridium septicum* empyema in an immunocompetent woman following operation for an incarcerated internal hernia. The patient was successfully treated with pleural decortication and an extended course of postoperative antibiotics. This is the first report of such an infection in the medical literature.

## 1. Introduction

*Clostridium septicum* is an anaerobic, spore-forming gram-positive bacillus. The organism is ubiquitous and is readily found in soil as well as in the gastrointestinal tract of humans [1]. *C. septicum* causes disease in nonhuman mammals and birds, and it was recognized historically as one of the causes of gas gangrene arising from battlefield injuries [1]. In the 1960s and 1970s, it became apparent that there was a group of individuals who were uniquely susceptible to infection via nontraumatic routes, namely, those with hematologic malignancies [2–4]. Over the ensuing decades, the number of cases attributable to other malignancies, particularly colonic neoplasms, has increased dramatically such that the recommendation has been made that patients with spontaneous infections be screened for cancer [5]. Although nontraumatic infections in normal hosts do occur, they would appear to be quite rare, based upon our review of the available literature. Herein we report on a monomicrobial pleural space infection with *C. septicum* in an otherwise normal host.

## 2. Case Report

**Figure 1:**
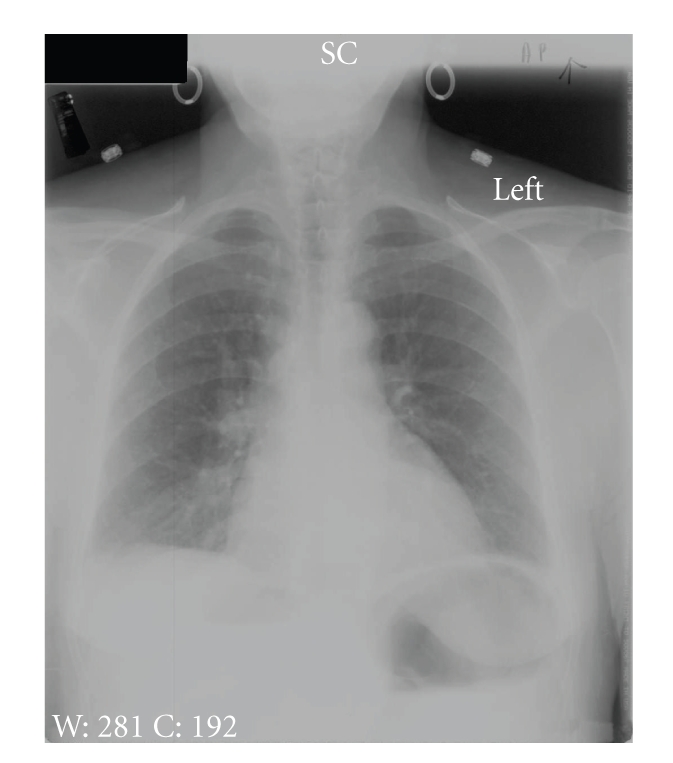
Patient's initial AP chest radiograph. Increased markings are noted in the right lower lobe, along with a possible small right pleural effusion.

**Figure 2:**
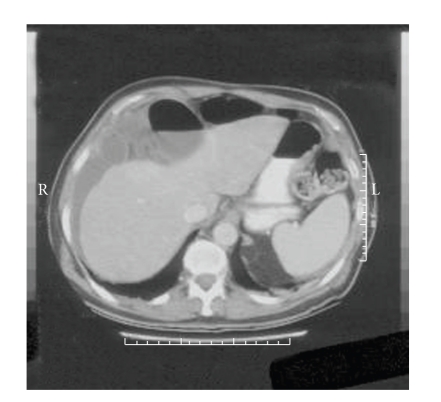
Representative section from the patient's abdominal CT scan. Dilated, fluid-filled loops of small and large bowel are noted anterior and superior to the liver, along with free fluid.

The patient is a 68-year-old woman who presented to the hospital with less than 24 hours of right upper-quadrant abdominal pain and nausea. She had a past history of alcohol and tobacco abuse, although she had been abstinent from both for five years at the time of presentation. No HIV risk factors were identified. She did not have a fever, and she had moderate abdominal tenderness on examination. Her chest X-ray was interpreted as showing a right lower-lobe infiltrate (Figure 1), and she received intravenous ceftriaxone and azithromycin for suspected community-acquired pneumonia. Because of persistent pain, an abdominal ultrasound was performed, which was negative for gallbladder wall thickening, stones, or pericholecystic fluid. A hepatobiliary iminodiacetic acid (HIDA) scan was also normal. The antibiotics were continued. The third day in the hospital, her abdomen became more diffusely tender and she developed atrial fibrillation with a rapid ventricular response. Her heart rate was controlled with intravenous diltiazem. An abdominal CT scan was performed, and it revealed multiple dilated loops of bowel anterior and superior to the liver, with associated free fluid (Figure 2). Laboratory studies demonstrated a leukocytosis of 13,000 white blood cells/mm^3^ with a left shift and two-dimensional echocardiography revealed a moderate pericardial effusion, without evidence of hemodynamic compromise. She was taken to the operating room, where she was found to have an internal hernia, with an ischemic loop of ileum incarcerated between the liver and the diaphragm. The hernia was reduced, and the ischemic segment of bowel was resected. She also underwent a cholecystectomy during the same procedure. Ciprofloxacin and metronidazole were administered postoperatively.

On the first postoperative day, control of her atrial fibrillation worsened. An echocardiogram was repeated, revealing an increase in size of the pericardial effusion. The cardiothoracic surgical service was consulted, and two days later she was taken back to the operating room and underwent drainage of the effusion and construction of a pericardial window. The pericardial fluid contained 2 white blood cells/*μ*L, with a manual differential of 33% polymorphonuclear leukocytes, 44% lymphocytes, and 23% monocytes. The total protein was 2.9 g/dL and the glucose was 131 mg/dL. Microbiologic stains of the pericardial fluid were negative for bacterial, fungal, and acid-fast organisms, and cultures were ultimately negative as well. She improved following the procedure and was discharged home 6 days later, on no antimicrobial therapy. 

**Figure 3:**
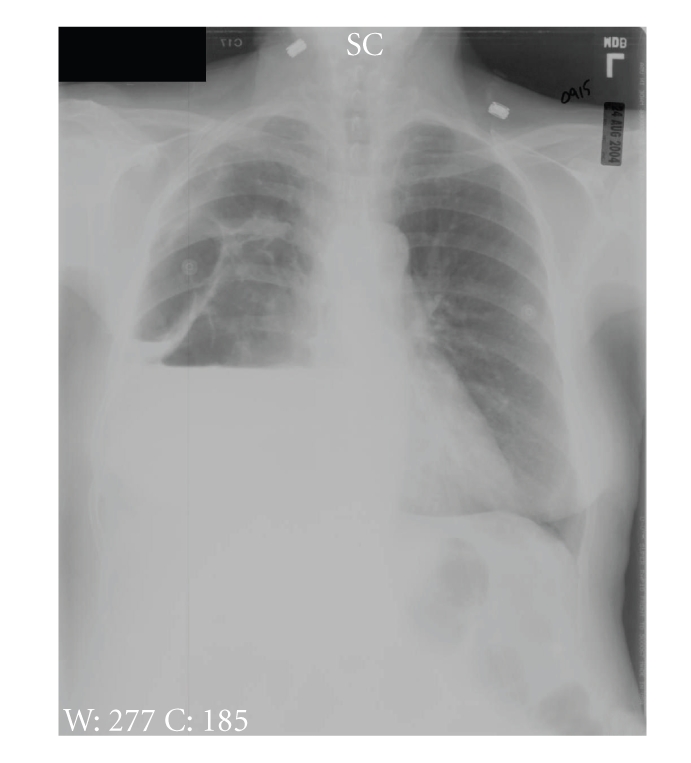
PA chest radiograph from the patient's readmission to the hospital. There is a large right pleural effusion, with an air- and fluid-filled structure noted laterally.

**Figure 4:**
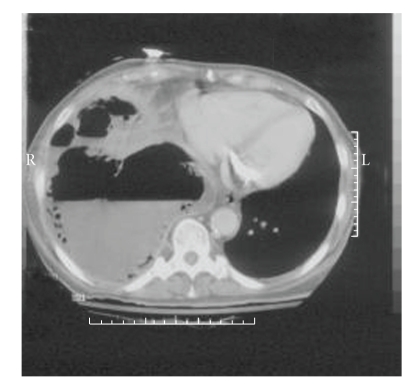
Representative section from the patient's chest CT scan, performed during the second admission. Most of the right chest is taken up by multiple collections of fluid and gas. Note the leftward deviation of the mediastinal structures.

The patient returned to the hospital 19 days later due to a complaint of worsening shortness of breath. Examination showed her to have a low-grade fever of 38°C and diminished breath sounds in the right chest. She had a white blood cell count of 24,500/mm^3^ with 18% band forms. The remainder of her CBC was normal. Her chemistries and electrolytes were unremarkable, except for a depressed albumin of 1.8 g/dL. Blood cultures were obtained and were negative. Her chest X-ray showed a right upper-lobe infiltrate with a large associated effusion (Figure 3). She was started on piperacillin/tazobactam and azithromycin, intravenously. Review of her chest X-ray suggested that she likely had several intrathoracic fluid collections. A CT scan of the chest was performed, demonstrating a large, multiloculated fluid collection in the right chest, with multiple air-fluid levels (Figure 4). There was a mass effect on the mediastinal structures. It could not be determined whether the fluid was contained above or below the diaphragm, so the patient was taken to the operating room, where an initial retroperitoneal approach showed that the fluid was in fact in the thoracic cavity. She then underwent thoracoscopy, which immediately encountered 1000 mL of thin, brown fluid and gas. Decortication was attempted via the thoracoscope, but ultimately was completed via open thoracotomy. Gram stain of the pleural fluid was negative, but anaerobic cultures yielded *Clostridium septicum*. The organism was identified using the RapID ANA II system (Remel Products, Lenexa, KS, USA). The organism did not produce *β*-lactamase. No additional susceptibility studies were performed. There was no growth in aerobic cultures. Her antibiotics were changed to aqueous penicillin G, 3 million units IV every four hours, and she was discharged home from the hospital on the sixth postoperative day to complete four weeks of parenteral antibiotic therapy at home. She made a full recovery, and a colonoscopy, performed to rule out a colonic neoplasm, was negative. She is still doing well, now over five years later.

## 3. Discussion

In an earlier review, Spagnuolo and Payne reported that spontaneous chest infections with *Clostridia* spp. do occur in patients without trauma or apparent neoplasm [6]. The majority of reviewed cases, however, still had some form of immune system compromise or other long-term chronic illnesses. None of the patients in that small review were infected with *C. septicum*. Two later reviews reiterated that only five species of *Clostridium*, namely, *C. perfringens*, *C. sordellii*, *C. sporogenes*, *C. paraputrificum*, and *C. bifermentans*, had been isolated as sole pathogens in cases of pleuropulmonary infection [7, 8]. A more general review of the microbiology of anaerobic empyema found that *Clostridium* spp. constituted approximately four percent of all isolated organisms (7/161) [9]. Most of these were not isolated in pure culture, and, again, *C. septicum* was not identified from any patient's infection. 

It would appear likely that our patient's infection arose from her compromised gastrointestinal tract. Whether this was from direct spread across the diaphragm or from hematogenous dissemination is not clear. In their review, Patel and Mahler [8] noted that a small but important subset of patients likely acquired their infection due to gut pathology. The observation that there are a significant number of cases of *C. septicum* infection associated with colon cancer may have more to do with intestinal damage than from any immunosuppressive effect of the neoplasm itself. Our patient was not found to have cancer. 

The mortality rate of *Clostridium* infections in the chest was estimated at 30% in one review, although the authors note that attributable mortality and mortality with appropriate antibiotics and drainage is significantly lower [8]. Given that most patients with nontraumatic infections with *C. septicum* appear to be immunocompromised, are there any specific factors which we can infer may be protective? The primary virulence factor of *C. septicum* appears to be alpha toxin, which exhibits necrotizing and hemolytic activities in vitro and in animal models [1, 10]. Thus, Johnson et al. [11] reported on the antibody response to alpha toxin in seven survivors of *C. septicum* bacteremia. Two of their patients were considered normal hosts, who likely became infected as the result of a surgical procedure. Only one of these, who presented with myonecrosis and gas gangrene, developed neutralizing antibodies. One of the immunocompromised patients in their series, an individual with occult cecal carcinoma, also developed antibodies to alpha toxin (not found to be neutralizing). None of the other immunocompromised patients developed antibodies, yet they nonetheless survived. Furthermore, antibodies to alpha toxin do not appear to be present in the general population, nor in immunocompromised individuals without infection. Indeed, survival thus appears to be more dependent upon appropriate antimicrobial therapy and surgical drainage than on the development of a humoral immune response. 

In summary, our patient was unusual in several ways. First, she was not found to have any immune compromise which would have led to an increased susceptibility to infection. Second, she developed an infection in a previously unreported site. This case adds to the available clinical information and body of literature surrounding this infrequently encountered, yet, nevertheless, medically important pathogen.
